# Supplementary material for: Dynamics of Florida milk production and total phosphate in Lake Okeechobee
Source: PLoS One. 2021 Aug 5;16(8):e0248910. doi: 10.1371/journal.pone.0248910 (PMC8341533; doi:10.1371/journal.pone.0248910)
Supplement: S1 Appendix — (PDF) [file pone.0248910.s001.pdf]

S1 Appendix

Empirical mode decomposition. Fig 5 shows the empirical mode decompositions of the milk production and lake phosphorus time series.

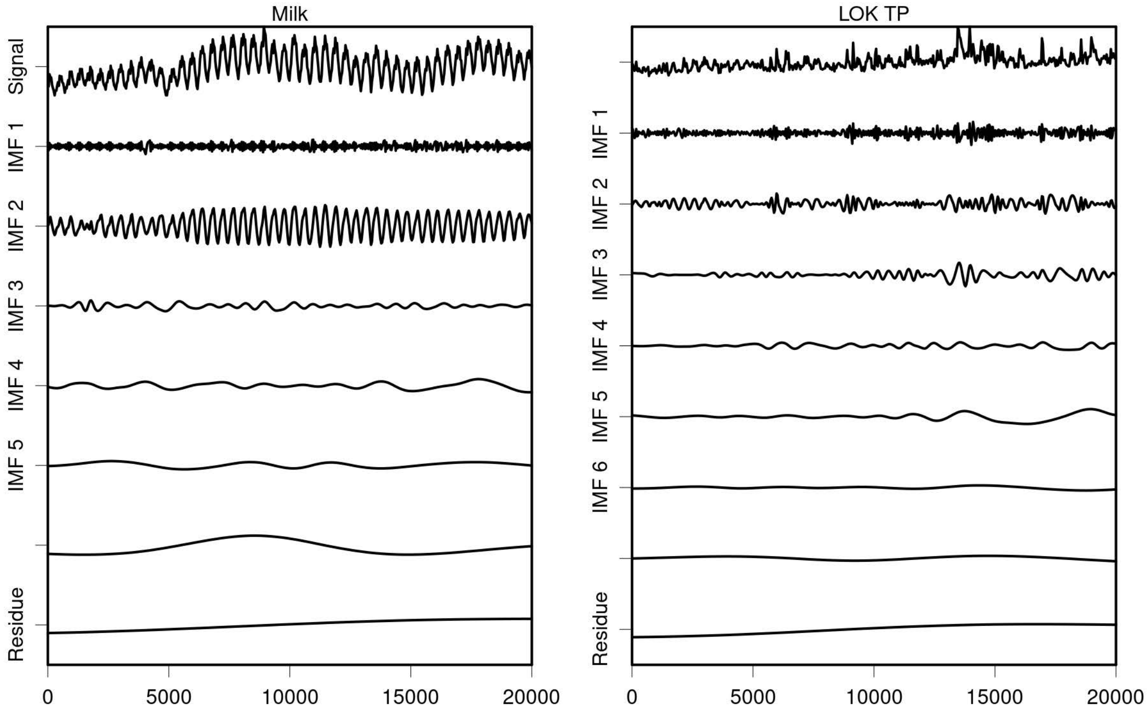

Fig 5. Empirical mode decompositions of milk production and lake phosphorus time series.

El-Niño Southern Oscillation and milk production

To assess links between interannual dynamics of milk production and exogenous forcing, we explore the hypothesis that positive ENSO phases, El-Niño conditions, can result in increased milk productivity. The hypothesis i s based on two underlying f acts: First, that El-Niño conditions tend to produce cooler and wetter conditions i n Florida [1] , and second, a negative linear relationship between Holstien milk production and mean daily temperature [2]. That is, Holstien milk production increases as temperatures moderate from 82°F to 72°F. We therefore expect a positive relationship between El-Niño conditions and milk production. To quantify the state of ENSO, we use the Multivariate ENSO Index (MEI) [3].

Interannual components of milk production and MEI are shown in Fig 6, where visually, there seems to be some correlation between milk production and MEI.

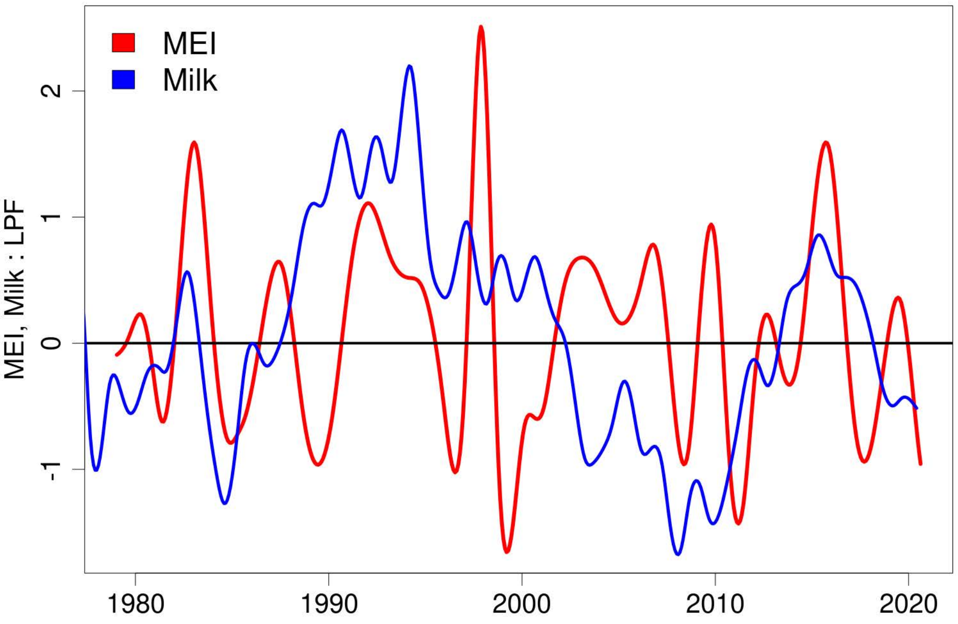

Fig 6. Interannual components of milk production and the Multivariate ENSO Index (MEI).

We assess links between these time series using CCM with results shown in Fig 7, indicating no significant link between ENSO state and milk production. Interestingly, the linear correlation is weak, but statistically significant with a p-value of 0.0019. In contrast, the CCM analysis finds a p-value of 0.32, indicating an insignificant relationship. The inappropriateness of a linear model is clear when time series are viewed in a scatter plot, Fig 8, clearly indicating nonlinear state dependence and a lack of dependence between MEI and milk production if state dependence is ignored.

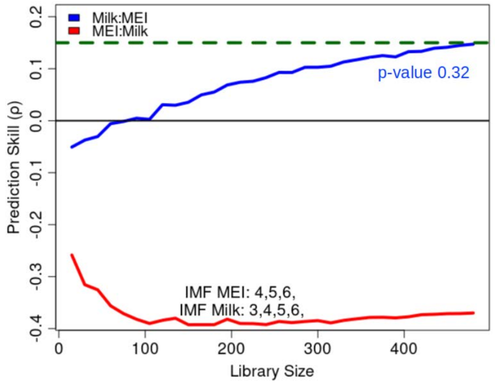

Fig 7. Convergent cross mapping between interannual components of milk production and MEI. Dashed horizontal line is linear cross correlation.

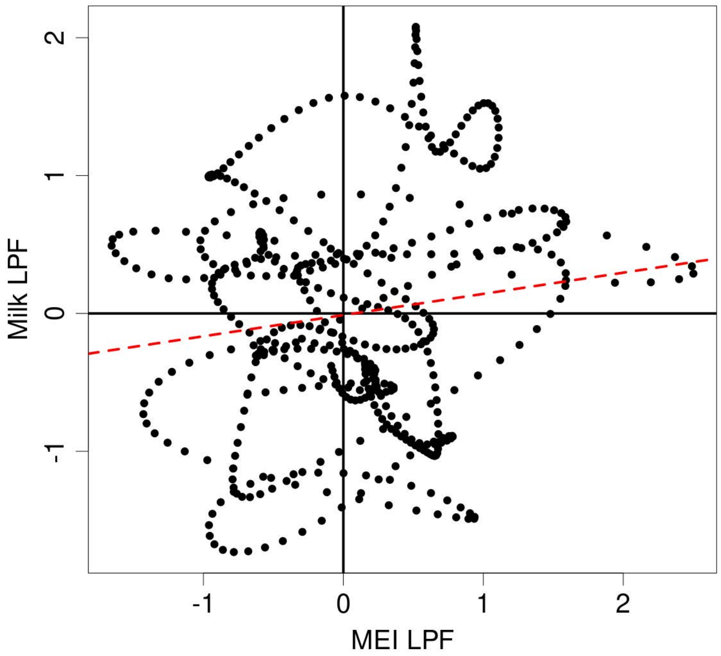

Fig 8. Interannual components of milk production vs. MEI. Dashed red line is the linear regression.

References

1. National Oceanic and Atmospheric Administration, National Weather Service. El Niño and its Effect on the Southeast. U.S. www.weather.gov/tae/enso.

2. West J. W., B. G. Mullinix, and J. K. Bernard (2003). Effects of Hot, Humid Weather on Milk Temperature, Dry Matter Intake, and Milk Yield of Lactating Dairy Cows. American Dairy Science Association, J. Dairy Sci. 86:232–242

3. National Oceanic and Atmospheric Administration, Physical Sciences Laboratory. Multivariate ENSO Index. psl.noaa.gov/enso/mei/.
