# Supplementary material for: Dynamics of Florida milk production and total phosphate in Lake Okeechobee
Source: PLoS One. 2021 Aug 5;16(8):e0248910. doi: 10.1371/journal.pone.0248910 (PMC8341533; doi:10.1371/journal.pone.0248910)
Supplement: Attachment — Submitted filename: Review-Response-1.pdf [file pone.0248910.s002.pdf]

PONE-D-21-07447

Dynamics of Florida milk production and total phosphate in Lake Okeechobee

Dear PLoS One, June 27, 2021

Thank you for the review of this manuscript. Response to review comments are found on the following pages. Journal Requirements are addressed in the attached Cover Letter.

Sincerely,

Joseph Park

Response to Reviewer #1

Thank you for providing this review. The comments serve to clarify and improve the presentation of the material. Please note that all comments are addressed explicitly with indented paragraphs below the reviewer comment.

Reviewer #1: Overall:
I thought the manuscript provided a novel application to assessing the causality of drivers of phosphorus dynamics in Lake Okeechobee using the EMD, EDM, and CCM approaches. My main concern is the article in its briefness fails to adequately aid the reader in following and interpreting their tautology and results. While I am familiar with the basics of EMD, EDM, and CCM, the average reader will not be, especially those involved in the science and management of Lake Okeechobee. In this system in particular, it is critical that the results and the logic of the inference is clear to the reader. It is not in this manuscript.

> Thank you for the critique and review of the manuscript. We have addressed all comments and believe the presentation is improved.

Introduction overall:
It would be useful for the reader in the “Contemporary Conditions and Restoration” or “Dynamical Perspective” sections to make it abundantly clear to the reader that your concern is with the loading of phosphorus to the lake and not the in-lake phosphorus concentration. While they are invariably linked, the legacy phosphorus in Lake Okeechobee is likely to have a larger impact on downstream conditions than the in-flow conditions. In-lake total phosphorus has increased dramatically in recent years and around hurricane events. Minimal decreases in in-flow concentrations are likely to have almost no effect in the short-term on the outflow conditions given the lake’s size, circulation, and immense storage of legacy phosphorus.

> Thank you for this perspective. We agree it is imperative to clearly communicate distinction between concentration and loading. We have corrected the erroneous use of "loading" where "concentration" is warranted.

Line 71-72: This is insufficient information to justify your choice of IMFs in subsequent analyses. The average reader will not be able to understand your methodological choices (lines 95-99) and their impacts without further information.

> Thank you for pointing out this deficiency. To address this, we have expanded the text providing additional information on physical relevance, and, to motivate the unfamiliar reader to consult the supporting references.

Line 76-77: This is again insufficient information for the average reader to understand your methodological choices (lines 95-99) and their impacts.

We have added a high-level technical description of EDM, and, have reworded the text suggesting introductory material for the reader seeking clarification of EDM.

Materials and methods:
Overall: There is no mention of El Niño in the methods at all, just the results.

This has been added to the Materials and methods.

Line 84-87: This is insufficient information to recreate your search query. Provide more information. Did you use all milk production in Florida? Or particular counties in the Lake Okeechobee watershed? Justify your choice.

While it would be preferrable to have data specific to the LOK watershed, that data was not available in a continously valued time series back to 1970. Milk production data are total monthly values for the state in millions of pounds. We have explicitly stated this in the text.

Line 88: Which stations? Siders and Havens (2020) shows and discusses how the DBHYDRO stations have changed in sampling scope over time. How do you assess whether these stations were reliable. Did all stations report across the whole time series or were they biased to particular inflows? In earlier years of the time series, the sampling was particularly sparse, how was this handled? Stations nearer to the Kissimmee River inflow are probably the only reliable stations to use to measure the inflow dynamics.

Thank you for this comment. As the reviewer notes, WQ sampling in LOK, and generally throughout the South Florida region is often an ad-hoc endeavour, where "stations" are ephemeral entries in a database rather than in the canonical sense, not sampled uniformly in time. Stations were selected based on criteria of 40 year period-of-record, and, subjected to manual QA inspection. The stations (L001, L003, L004, L007, L008) have been added to the text.

Line 91: How does this spline perform? What is the coverage of the original data? Given the changing sampling patterns of the DBHYDRO stations over time, how reliable is the spline at interpolating the information. Siders and Havens (2020) shows that the sampling is highly autocorrelated, how did you handle this in your interpolation.

The spline performs quite well, as it is applied to the 5-station average. Residuals range from -2.22e-16 to 8.33e-17. As noted above, the ad-hoc nature of sampling provides motivation for the station average with spline interpolation. This maintains the data-driven aspect of the analysis, where models and presumptions are eschewed.

More directly, the question might be: Is the the 5-station average representative of the overall phosphate concentration on monthly to decadal time scales? Given the

aggregated nature of the Milk production data, the sensible dynamics of the resultant aggregated phosphorus time series, and, the analytical results, on can argue that it does.

Regarding autocorrelation, this is not a concern for the spline. Indeed, one would expect it to benefit interpolation. It is however a concern in the subsequent analysis, and, we address that in the EDM analysis with a temporal "exclusion radius" as noted in the manuscript.

Line 88-91: Why choose in-lake stations at all if the focus is on the inflow phosphorus dynamics?

We use in-lake data since it constitutes the multidecadal period-of-record, and, is sampled at roughly the same spatial locations. We are not aware of such long-term, spatially stable data available in the upstream watershed. Further, the lake is presumed to be a significant source of "new" water for CERP.

Line 97: How did you determine they had interannual frequencies? If this is intrinsic to IMFs, then this should be clear earlier in the manuscript.

IMF frequencies are obtained from the Hilbert Transform (instantaneous frequencies). These are part of the Hilbert-Huang transform. We use the mean value of the instantaneous frequency of each IMF to represent the overall frequency, and thus inverse period, of the IMF. Intra-annual IMFs are deemed to have mean periods between 0.5 and 2 years. Interannual IMFs have periods greater than 3 years. This has been added to the text.

Results:
Line 131: There is only five decades in the data. I’m not sure how this is terribly surprising just given human population growth in the region. It seems that a remarkably high amount of time series would show the same trend when scaled.

The point is agreed. At least the part about anticipated coherence between human population and milk consumption/phosphate runoff. However, having five decades of phosphate measurements is, at least in my (J Park) opinion, rather remarkable. While the population increase is expected, the trend of milk production and phosphate concentration stabilising in the 21st Century are not while population growth in has continued. Further, the scaled nonlinear trends are nearly identical. That is, the overal *rate* of milk production and phosphate over 5 decades appears tightly coupled. While we have not examined other long-term data, it would be surprising to see such such close coherence in the rates over multi-decadal time scales. An interesting question for follow-on analysis.

Line 135: That data used was on in-lake concentrations not loadings, this statement is not accurate.

Thank you. Corrected.

Line 146-149: The phrasing on the CCM notation is exceptionally confusing. Please rephrase so the reader can clearly understand the take away.

Thank you for this important comment. This is often a point of confusion with CCM. The text has been replaced to clearly explain the nomenclature.

Line 151: The low-pass filter is not mentioned in the methods.

Thank you for noting this omission. We use the term low-pass filter as synomous with the removal of the highest frequency IMF. This has been explicitly stated.

Line 156-159: This is the first mention of El Niño. It is confusing.

Thank you for this comment. We have added text to provide the missing context, relating it to the analysis of El Niño in the appendix.

Line 160-162: Seasonal patterns in Lake Okeechobee have been discussed at length in the literature. This should be discussed.

Thank you for this comment. We have added a citation and mention of a possible link between seasonal wind/temperature and P/milk production.

Discussion:

Line 167: Citation?

Please note that this issue was discussed in the Introduction with citation. We simply restate it here to provide context for the conclusion.

Line 174-177: That data used was on in-lake concentrations not loadings, this statement is not accurate.

Corrected.

Figures:

Figures 2 and 3 can be merged.

> We respectfully submit that it is logical to maintain separation between these figures. Figure 2 represents time scale filtered versions of the data, with no transformations/scaling. Figure 3 transforms/scales the data. It also would create a single figure with 10 panels, which we deem excessive.

Figures 3 and 4 should be color-blind friendly.

> Corrected.
